# Supplementary material for: Expanding the landscape of aging via orbitrap astral mass spectrometry and tandem mass tag integration
Source: Nat Commun. 2025 May 22;16:4753. doi: 10.1038/s41467-025-60022-x (PMC12098839; doi:10.1038/s41467-025-60022-x)
Supplement: Supplementary file 1 — Supplementary Information [file 41467_2025_60022_MOESM1_ESM.pdf]

Supplementary Figures

a

Reagent	Reporter Ion Mass	Detected	Δmass
TMTpro–126	126.127726	126.1293	0.001574
TMTpro–127N	127.124761	127.1227	-0.002061
TMTpro–127C	127.131081	127.1311	0.000981
TMTpro–128N	128.128116	128.1254	-0.002716
TMTpro–128C	128.134436	128.1341	-0.000336
TMTpro–129N	129.131471		
TMTpro–129C	129.137791	129.1359	-0.001891
TMTpro–130N	130.134826		
TMTpro–130C	130.141146	130.1386	-0.002546
TMTpro–131N	131.138181	131.1411	0.002919
TMTpro–131C	131.144501		
TMTpro–132N	132.141536		
TMTpro–132C	132.147856	132.1459	-0.001956
TMTpro–133N	133.144891		
TMTpro–133C	133.151211	133.1491	-0.002111
TMTpro–134N	134.148246	134.1511	-0.002854
TMTpro–134C	134.154566		
TMTpro–135N	135.151601	135.1510	-0.000601

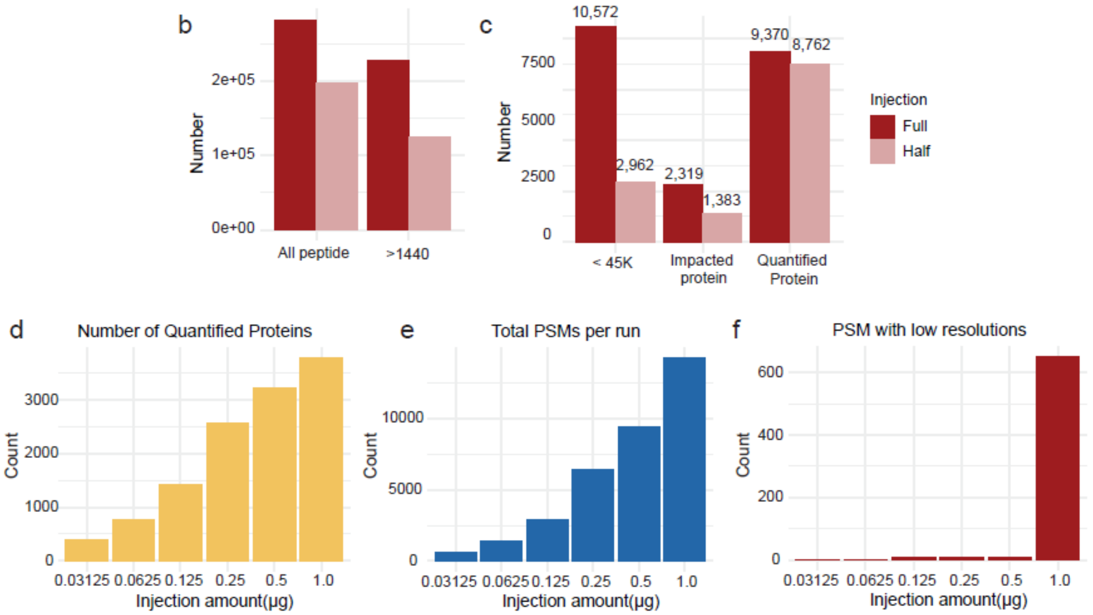

Supplementary Figure 1. Reducing injection material does not eliminate the ion saturation problem. (a) In the scan shown in Fig. 1b, twelve of the eighteen ions have mass differences to its nearest TMT reporter ions smaller than 0.003 Da and identified as TMT reporter ions. Those with smaller than 0.001 Da were highlighted in yellow. (b) Comparison of the total number of quantified peptides and peptides with an S/N greater than 1,440 across 18 channels between the original analysis and the analysis using half the injection material. (c) Comparison of peptides with low resolution, impacted proteins, and quantified proteins between the original analysis and the half-material analysis. (d) Number of quantified proteins, (e) total PSMs used for protein quantitation, and (f) PSMs with low resolution decrease with reduced injection material.

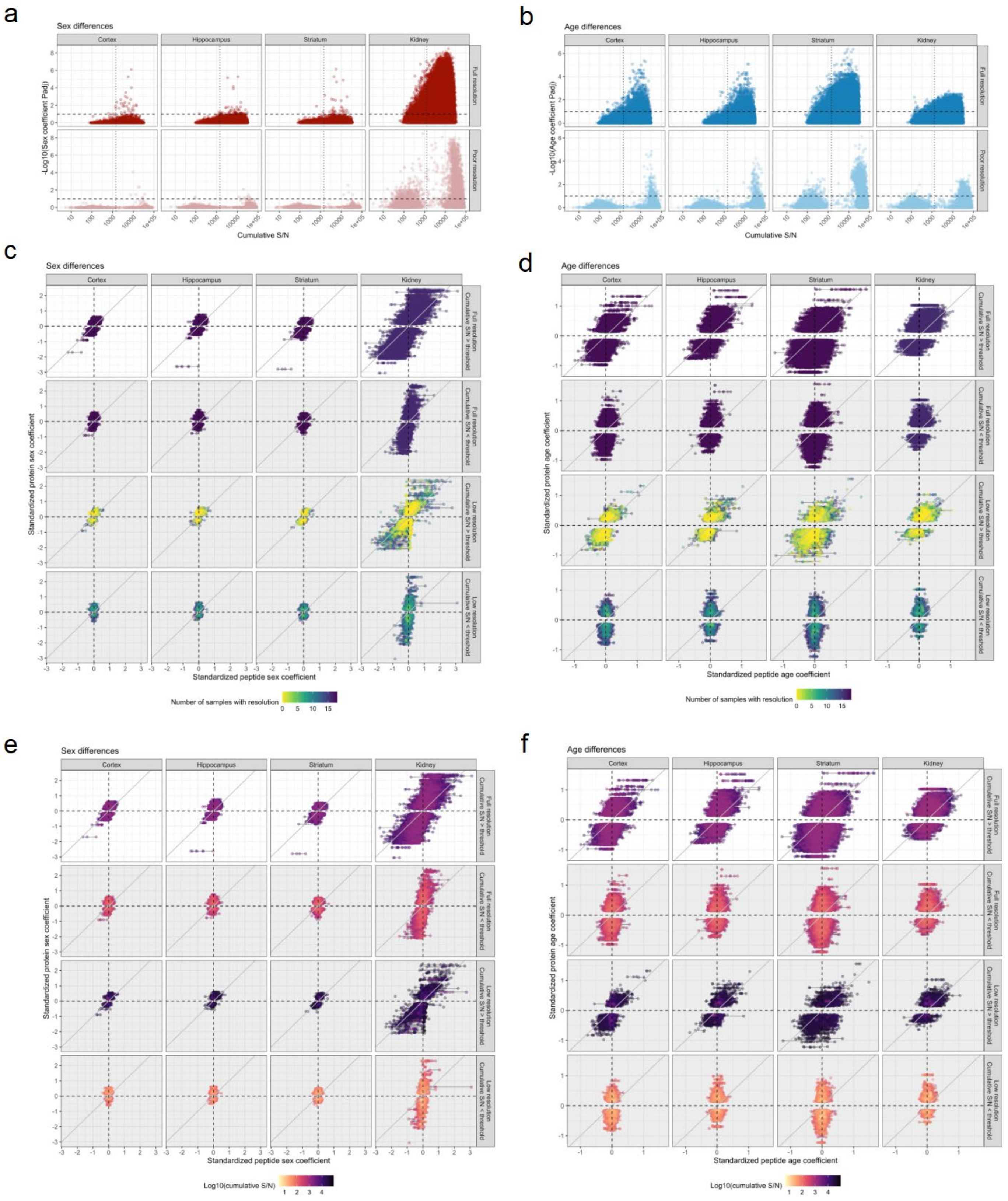

Supplementary Figure 2. Impact of resolution and S/N on detecting age- and sex-differences in peptides. Association between statistical significance of test by cumulative S/N for (a) sex and (b) age differences, stratified by full resolution status. Vertical dotted line represents the cumulative S/N threshold for peptide filtering. Standardized regression coefficients for protein measurements by standardized regression

coefficients from peptide measurements for (c, e) sex and (d, f) age, stratified by full resolution and cumulative S/N statuses. Peptides from the same protein are connected by lines. Points are colored by resolution (c, d) and cumulative S/N (e, f).

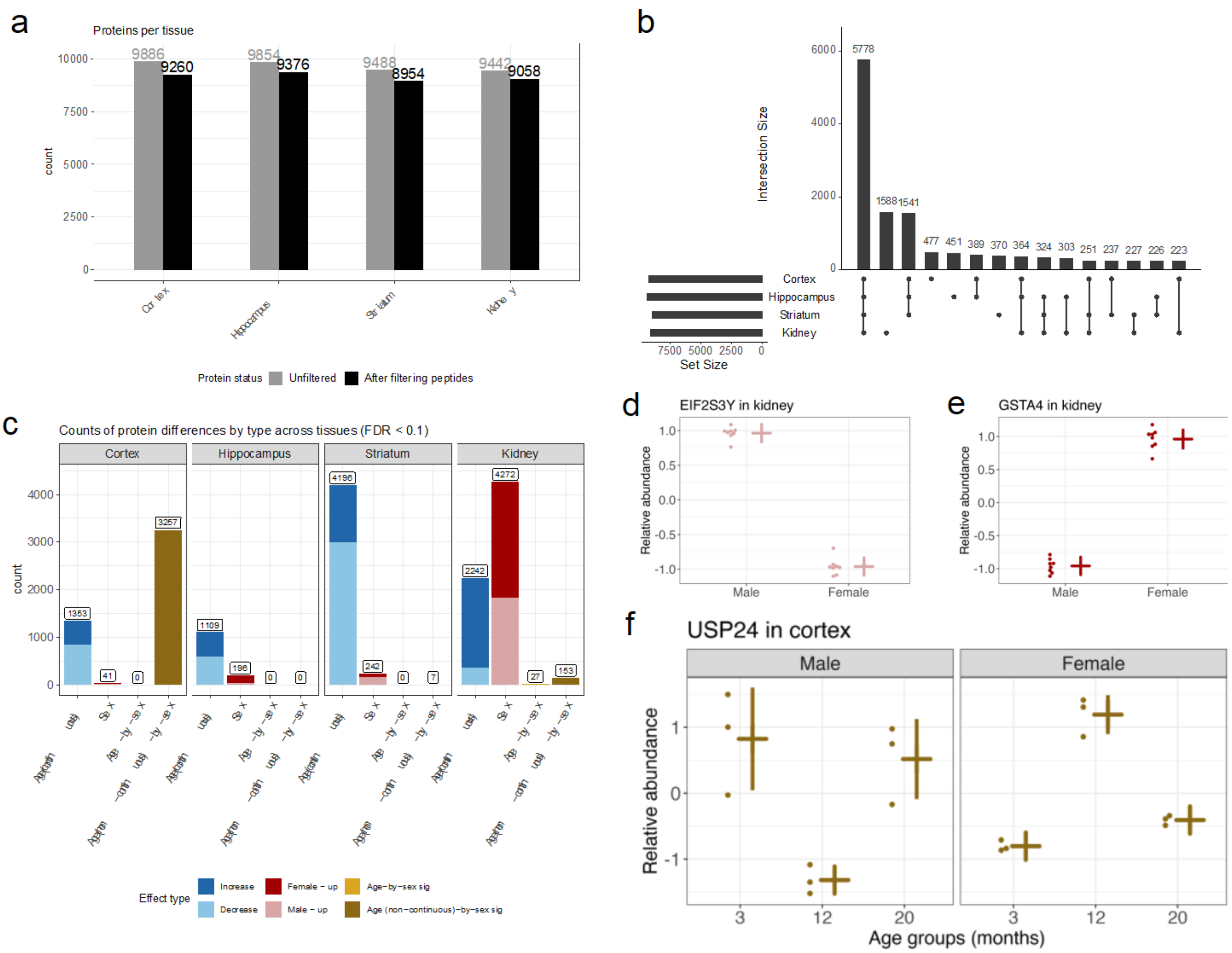

Supplementary Figure 3. Summaries of protein counts and significant differences (FDR < 0.1). (a) Counts of detected proteins by tissues, before and after peptide filtration. (b) Upset plot summarizing the number of proteins observed across the four tissues. (c) Counts of statistically significant (FDR < 0.1) differences in each tissue. Differences in individual proteins tested with F tests, followed by FDR adjustment. (d) EIF2S3Y had higher abundance in male kidneys. N = 8 biological replicates for both sexes. Mean ± SD indicated for each sex. (e) GSTA4 had higher abundance in female kidneys. N = 8 biological replicates for both sexes. Mean ± SD indicated for each sex. (f) USP24 had a non-continuous age-by-sex difference in cortex, characterized by flipped age-related abundance patterns between females and males. N = 3 biological replicates for each sex-by-age group. Mean ± SD indicated for each age-by-sex group.

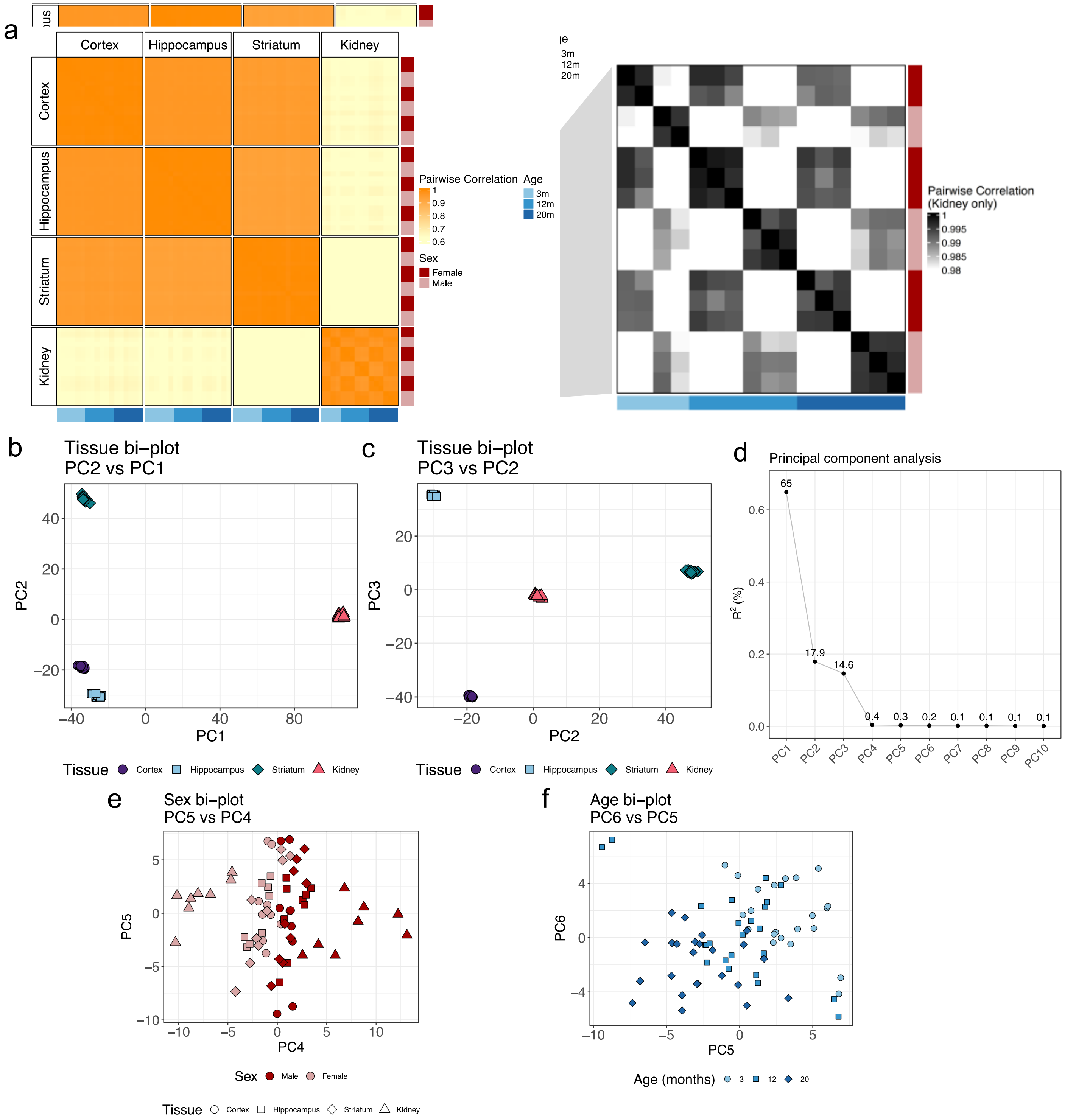

Supplementary Figure 4. Principal component analysis (PCA) reveals tissue, sex, and age as drivers of overall protein variation. (a) Heatmap of mouse-level correlation matrix based on the 5778 proteins observed across all four tissues. Columns and rows are ordered by sex and age groups to highlight potential contributions to correlation patterns. Kidney correlation matrix blown up to highlight the impact of sex on overall variation. Bi-plots of (b) PC2 by PC1 and (c) PC3 by PC2, highlighting tissue as the major driver of variation across proteins. (d) Scree plot of the PCA, describing the proportion of overall variation explained by each PC. (e) Bi-plot of PC5 by PC4, highlighting that sex is the primary driver of PC4. (f) Bi-plot of PC6 by PC5,

highlighting a continuous age effect across both PCs. PCA results for early development from Wang *et al.* 2024 and adulthood to geriatric age from Takasugi *et al.* 2024 are in Supplementary Figure 7.

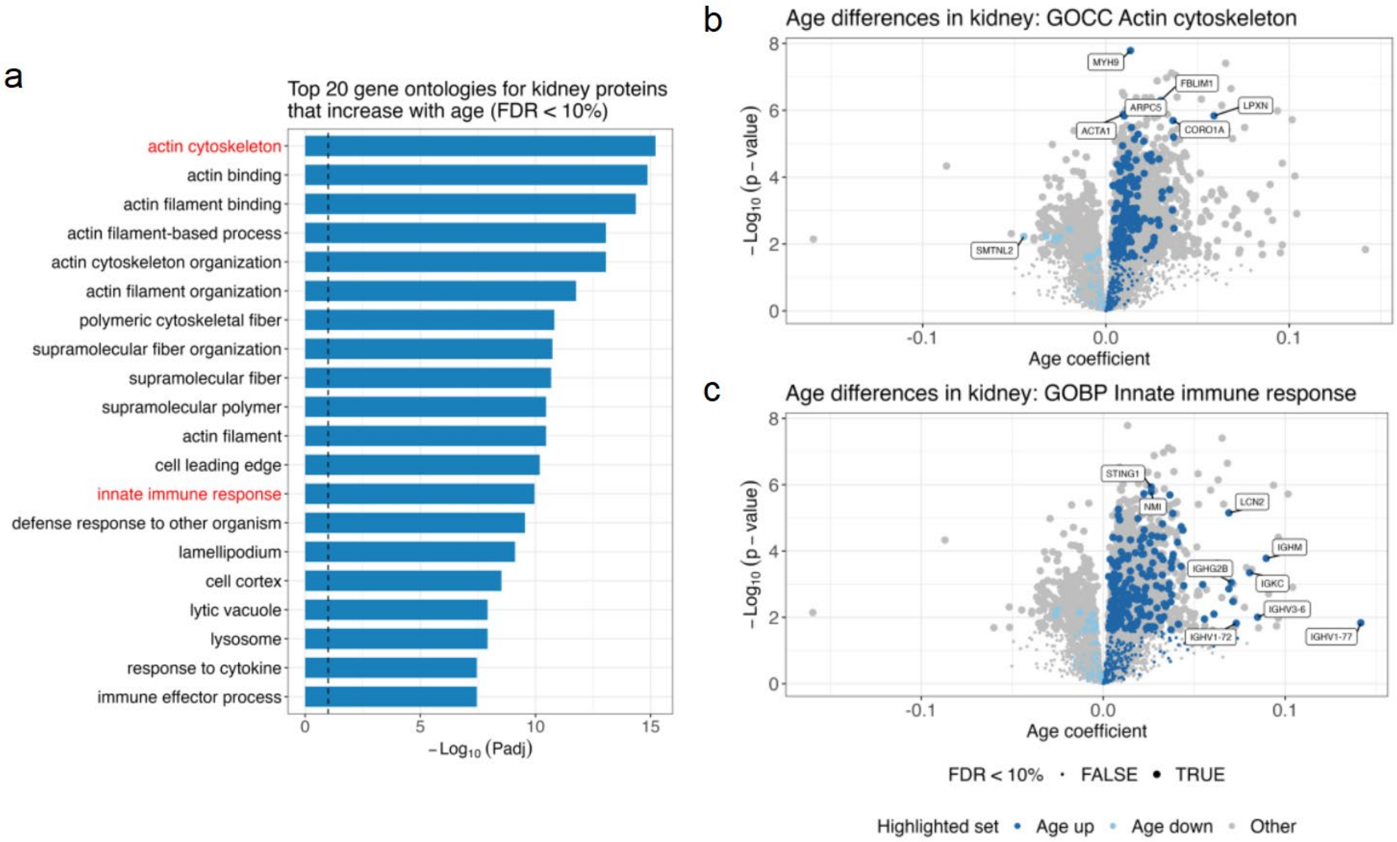

Supplementary Figure 5. Gene set enrichment results for proteins that increase in abundance with age in kidney, including actin cytoskeleton and innate immune response proteins. (a) Top 20 gene ontologies enriched in kidney proteins with increased abundance with age (FDR < 0.1). Enrichment analysis performed using hypergeometric tests, followed by FDR adjustment. Vertical dashed line represents a threshold of FDR < 0.1. Actin cytoskeleton and innate immune response gene sets are highlighted with red text, which are featured in Supplementary Fig. 5b-c. (b) Volcano plot of continuous age differences in kidney with actin cytoskeleton proteins highlighted. Age differences in individual proteins tested with F tests, followed by FDR adjustment.
(c) Volcano plot of continuous age differences in kidney with innate immune response proteins highlighted. Age differences in individual proteins tested with F tests, followed by FDR adjustment.

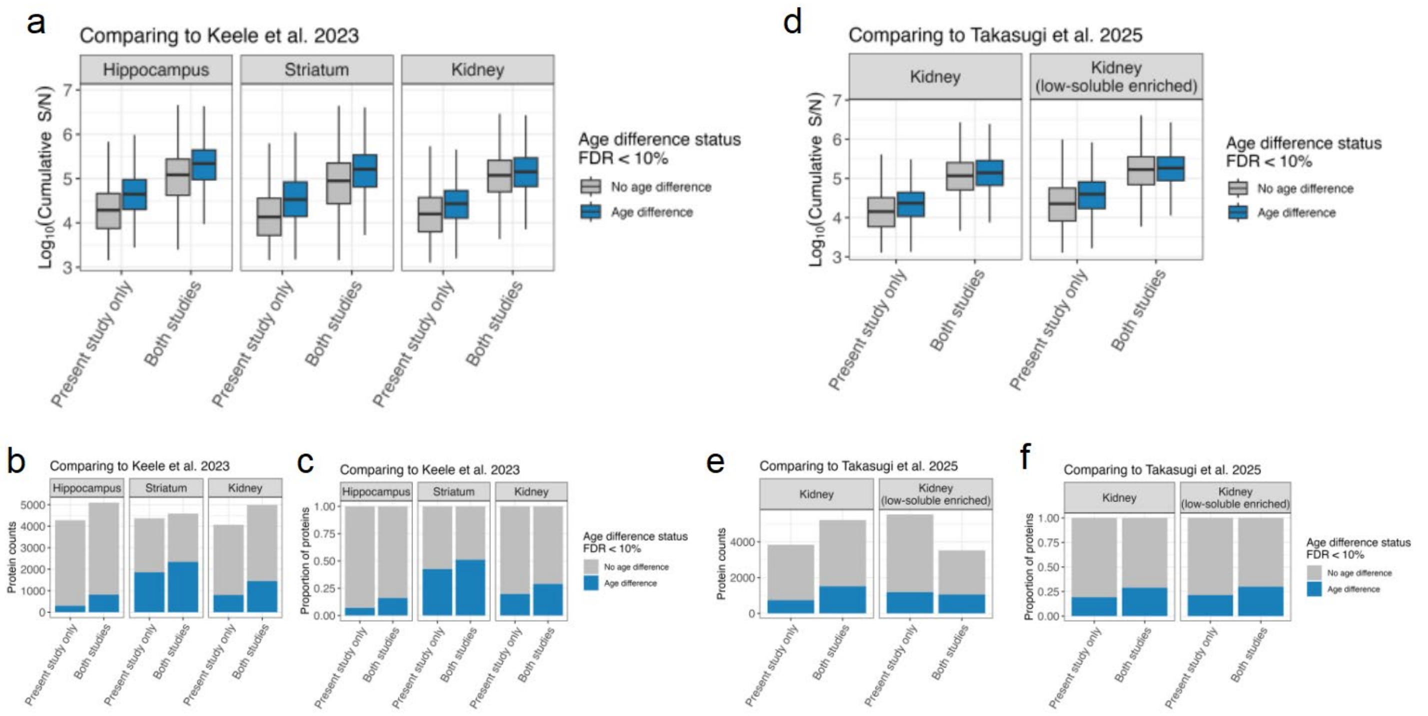

Supplementary Figure 6. Breakdown of protein age difference detection based on whether they were observed in the present study only or in the present study and Keele *et al.* 2023 or Takasugi *et al.* 2025. Age differences in individual proteins tested with F tests, followed by FDR adjustment in each study. (a) Boxplots of cumulative S/N across proteins, categorized on whether they were observed in only the present study or both present study and Keele *et al.* 2023. (b) Count of proteins with and without significant age differences, categorized on whether they were observed in only the present study or both present study and Keele *et al.* 2023. (c) Proportion of proteins with and without significant age differences, categorized on whether they were observed in only the present study or both present study and Keele *et al.* 2023. (d) Boxplots of cumulative S/N across proteins, categorized on whether they were observed in only the present study or both present study and Takasugi *et al.* 2025. (e) Count of proteins with and without significant age differences, categorized on whether they were observed in only the present study or both present study and Takasugi *et al.* 2025. (f) Proportion of proteins with and without significant age differences, categorized on whether they were observed in only the present study or both present study and Takasugi *et al.* 2025.

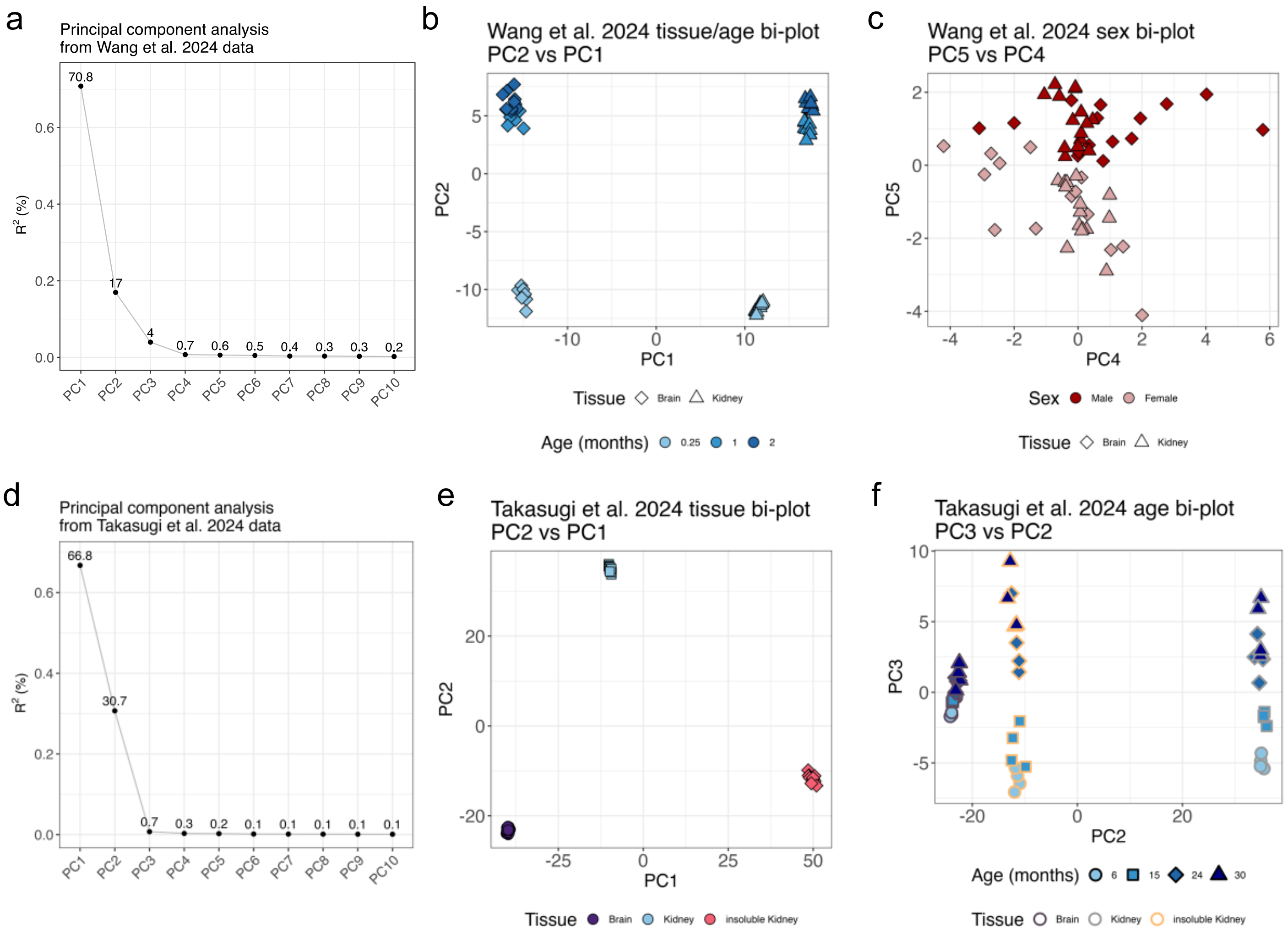

Supplementary Figure 7. Principal component analysis (PCA) reveals age as driver of overall protein variation in early development and adulthood to geriatric age. (a) Scree plot of the PCA, describing the proportion of overall variation explained by each PC, for Wang *et al.* 2024 data. (b) Bi-plot of PC2 by PC1, highlighting tissue as the primary driver of PC1 and age as the primary driver of PC2, for Wang *et al.* 2024 data. (c) Bi-plot of PC5 by PC4, highlighting sex as the primary driver of PC5, for Wang *et al.* 2024. (d) Scree plot of the PCA, describing the proportion of overall variation explained by each PC, for Takasugi *et al.* 2024 data. (e) Bi-plot of PC2 by PC1, highlighting tissue as the primary driver of PC1 and PC2, for Takasugi *et al.* 2024 data. (f) Bi-plot of PC3 by PC2, highlighting age as the primary driver of PC3, for Takasugi *et al.* 2024. PCA results for adulthood aging from the present study is in Supplementary Fig. 4.

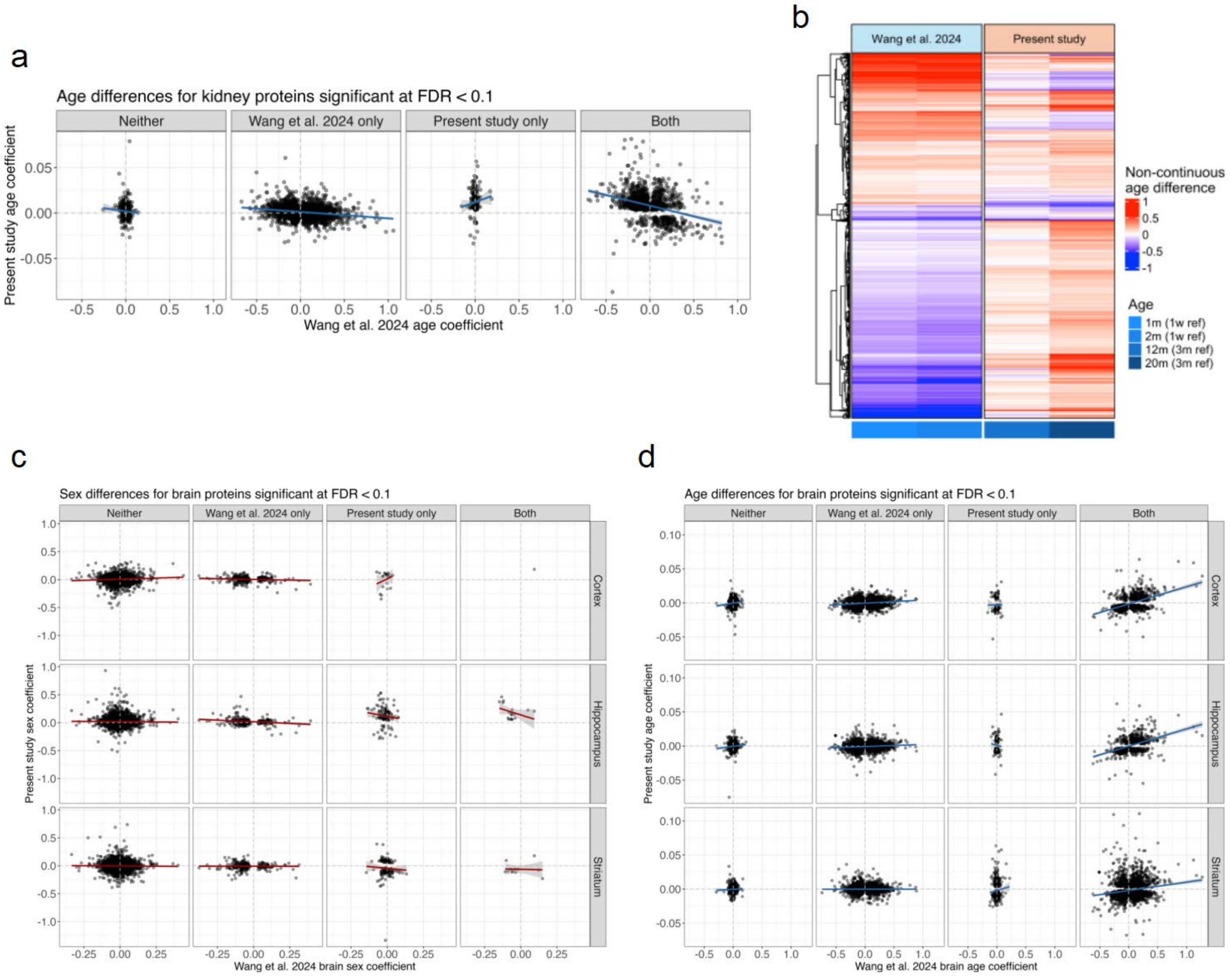

Supplementary Figure 8. Comparison to early development protein data reveals discordant aging patterns from adulthood depending on tissue. (a) Comparison of age difference coefficients for kidney between the present study and Wang *et al.* 2024. Best fit line from regressing age coefficients from the present study on those from Wang *et al.* 2024 ± SE included for reference. Age differences for individual proteins tested with F tests, followed by FDR adjustment in each study. (b) Heatmap of non-continuous kidney age difference coefficients that were significant (FDR < 0.1) in both the present study and Wang *et al.* 2024. Age differences for individual proteins tested with F tests, followed by FDR adjustment in each study. (c) Comparison of sex difference coefficients between three brain tissues from the present study and general brain tissue from Wang *et al.* 2024. Best fit line from regressing sex coefficients from the present study on those from Wang *et al.* 2024 ± SE included for reference. Sex differences for individual proteins tested with F tests, followed by FDR adjustment in each study. (d) Comparison of continuous age difference coefficients between three brain tissues from the present study and general brain tissue from Wang *et al.* 2024. Best fit line from regressing age coefficients from the present study on those from Wang *et al.* 2024 ± SE included for reference. Age differences for individual proteins tested with F tests, followed by FDR adjustment in each study.
