# Supplementary material for: Expanding the landscape of aging via orbitrap astral mass spectrometry and tandem mass tag integration
Source: Nat Commun. 2025 May 22;16:4753. doi: 10.1038/s41467-025-60022-x (PMC12098839; doi:10.1038/s41467-025-60022-x)
Supplement: Supplementary file 2 — Description of Additional Supplementary Files [file 41467_2025_60022_MOESM2_ESM.pdf]

Supplementary Data Legends

Supplementary Data 1. Mouse sample information. Spreadsheets linking TMT tag to mouse sample Information (age group, sex, individual identifier) for each tissue.

Supplementary Data 2. Protein signal-to-noise information. Spreadsheets providing quantitative abundance (signal-to-noise) data for all TMT tags for each protein in each tissue. Additional information includes gene symbols and ENSEMBL genes and protein identifiers.

Supplementary Data 3. Association summary statistics for individual tissues. Spreadsheet providing results from statistical testing of differences in protein abundance based on sex, continuous age, non-continuous age, and the interaction between sex and age, for each tissue. Statistical testing of sex and age differences in individual proteins was performed using F tests, followed by FDR adjustment.

Supplementary Data 4. GSEA results from cortex. Gene sets were defined based on statistically significant age or sex difference and the direction or type of difference pattern (Supplementary Table 4). Enrichment testing was performed using hypergeomtric tests, followed by FDR adjustment.

Supplementary Data 5. GSEA results from hippocampus. Gene sets were defined based on statistically significant age or sex differences (Supplementary Table 4). Enrichment testing was performed using hypergeomtric tests, followed by FDR adjustment.

Supplementary Data 6. GSEA results from striatum. Gene sets were defined based on statistically significant age or sex differences (Supplementary Table 4). Enrichment testing was performed using hypergeomtric tests, followed by FDR adjustment.

Supplementary Data 7. GSEA results from kidney. Gene sets were defined based on statistically significant age or sex differences (Supplementary Table 4). Enrichment testing was performed using hypergeomtric tests, followed by FDR adjustment.

Supplementary Data 8. Association summary statistics for joint modeling of brain tissues. Spreadsheet providing results from statistical testing of differences in protein abundance based on sex, continuous age, non-continuous age, and the interaction between sex and age, jointly across brain tissues. Statistical testing of sex and age differences in individual proteins was performed using F tests, followed by FDR adjustment.
