# Supplementary material for: Expanding the landscape of aging via orbitrap astral mass spectrometry and tandem mass tag integration
Source: Nat Commun. 2025 May 22;16:4753. doi: 10.1038/s41467-025-60022-x (PMC12098839; doi:10.1038/s41467-025-60022-x)
Supplement: Supplementary file 11 — Reporting Summary [file 41467_2025_60022_MOESM11_ESM.pdf]

nature portfolio

Corresponding author(s): TIAN ZHANG
Last updated by author(s): Apr 20, 2025

Reporting Summary

Nature Portfolio wishes to improve the reproducibility of the work that we publish. This form provides structure for consistency and transparency in reporting. For further information on Nature Portfolio policies, see our Editorial Policies and the Editorial Policy Checklist.

Statistics

For all statistical analyses, confirm that the following items are present in the figure legend, table legend, main text, or Methods section.

n/a	Confirmed	
☐	☒	The exact sample size (*n*) for each experimental group/condition, given as a discrete number and unit of measurement
☐	☒	A statement on whether measurements were taken from distinct samples or whether the same sample was measured repeatedly
☐	☒	The statistical test(s) used AND whether they are one- or two-sided<br>*Only common tests should be described solely by name; describe more complex techniques in the Methods section.*
☐	☒	A description of all covariates tested
☐	☒	A description of any assumptions or corrections, such as tests of normality and adjustment for multiple comparisons
☐	☒	A full description of the statistical parameters including central tendency (e.g. means) or other basic estimates (e.g. regression coefficient) AND variation (e.g. standard deviation) or associated estimates of uncertainty (e.g. confidence intervals)
☐	☒	For null hypothesis testing, the test statistic (e.g. *F*, *t*, *r*) with confidence intervals, effect sizes, degrees of freedom and *P* value noted<br>*Give P values as exact values whenever suitable.*
☒	☐	For Bayesian analysis, information on the choice of priors and Markov chain Monte Carlo settings
☒	☐	For hierarchical and complex designs, identification of the appropriate level for tests and full reporting of outcomes
☐	☒	Estimates of effect sizes (e.g. Cohen's *d*, Pearson's *r*), indicating how they were calculated

Our web collection on statistics for biologists contains articles on many of the points above.

Software and code

Policy information about availability of computer code

Data collection	We developed a C# program to extract the resolution of each TMT reporter ion in each scan. This program is available on figshare (https://doi.org/10.6084/m9.figshare.28016501).
Data analysis	All processed data, including supplementary tables, and the R code used for statistical analyses and generating the reported findings are publicly available on figshare (https://doi.org/10.6084/m9.figshare.28016501). All the data analysis in the Method section were performed in R (v4.4.3), and the R packages used include tidyverse (v2.0.0), readxl (v1.4.4), writexl (v1.5.2), clusterProfiler (v4.14.6), DOSE (v4.0.0), ggdist (v3.3.2), ComplexHeatmap (v2.22.0), circlize (v0.4.16), ggrepel (v0.9.6), UpSetR (v1.4.0), pcaMethods (v1.98.0) and ggsci (v3.2.0).

For manuscripts utilizing custom algorithms or software that are central to the research but not yet described in published literature, software must be made available to editors and reviewers. We strongly encourage code deposition in a community repository (e.g. GitHub). See the Nature Portfolio guidelines for submitting code & software for further information.

Data

Policy information about availability of data

All manuscripts must include a data availability statement. This statement should provide the following information, where applicable:

- Accession codes, unique identifiers, or web links for publicly available datasets
- A description of any restrictions on data availability
- For clinical datasets or third party data, please ensure that the statement adheres to our policy

The mass spectrometry proteomics data have been deposited to the ProteomeXchange Consortium via the PRIDE partner repository with the dataset identifier PXD058160 and 10.6019/PXD058160. Log in to the PRIDE website using the following details: Project accession: PXD058160; Token: TMDUSpfYvdKf. Alternatively, reviewers can access the dataset by logging in to the PRIDE website using the following account details: Username: reviewer_pxd058160@ebi.ac.uk;Password: fko8eHs1nW8Q. All processed data and R code to perform statistical analyses and generate the reported findings are publicly available at figshare (https://doi.org/10.6084/m9.figshare.28016501).

Research involving human participants, their data, or biological material

Policy information about studies with human participants or human data. See also policy information about sex, gender (identity/presentation), and sexual orientation and race, ethnicity and racism.

Reporting on sex and gender	Study did not involve human participants.
Reporting on race, ethnicity, or other socially relevant groupings	Study did not involve human participants.
Population characteristics	Study did not involve human participants.
Recruitment	Study did not involve human participants.
Ethics oversight	Study did not involve human participants.

Note that full information on the approval of the study protocol must also be provided in the manuscript.

Field-specific reporting

Please select the one below that is the best fit for your research. If you are not sure, read the appropriate sections before making your selection.

☒ Life sciences ☐ Behavioural & social sciences ☐ Ecological, evolutionary & environmental sciences

For a reference copy of the document with all sections, see nature.com/documents/nr-reporting-summary-flat.pdf

Life sciences study design

All studies must disclose on these points even when the disclosure is negative.

Sample size	18 mice were included with a tissue sample collected from four tissues per animal. The sample size was partly determined by the limits of the TMT 18plex technology. Prior studies had shown balanced study designs of this size were sufficient for detecting large numbers of age and sex differences.
Data exclusions	Kidney data from two mice were filtered out based on mass spectrometry QC analysis.
Replication	The study design included three biological replicates per sex and age group (3 age groups were surveyed). Summary statistics of age and sex differences were then compared to other studies for validation.
Randomization	Mice of specified sex and age groups were ordered from NIA. Animals were not assigned to an exposure/treatment group, so randomization concerns do not apply.
Blinding	The investigator was blinded to the selection of specific mice from NIA.

Reporting for specific materials, systems and methods

We require information from authors about some types of materials, experimental systems and methods used in many studies. Here, indicate whether each material, system or method listed is relevant to your study. If you are not sure if a list item applies to your research, read the appropriate section before selecting a response.

Materials & experimental systems

n/a	Involved in the study	
x		Antibodies
x		Eukaryotic cell lines
x		Palaeontology and archaeology
	x	Animals and other organisms
x		Clinical data
x		Dual use research of concern
x		Plants

Methods

n/a	Involved in the study	
x		ChIP-seq
x		Flow cytometry
x		MRI-based neuroimaging

Animals and other research organisms

Policy information about studies involving animals; ARRIVE guidelines recommended for reporting animal research, and Sex and Gender in Research

Laboratory animals	C57BL/6JN mice were used. Results were compared to external studies of other sub-strains of C57BL/6.
Wild animals	Study did not involve wild animals.
Reporting on sex	Study included balanced numbers of male and female mice (nine each, evenly distributed across three age groups). Sex was a factor of interest, thus incorporated into the analysis in terms as either a covariate or the tested factor. Sex-specific results are reported and made available.
Field-collected samples	Study did not involve samples collected from the field.
Ethics oversight	No ethical approval or guidance was required because mice tissue samples were ordered from the NIA where the animal handling was performed upstream of this study.

Note that full information on the approval of the study protocol must also be provided in the manuscript.

Plants

Seed stocks	This study did not involve plants.
Novel plant genotypes	This study did not involve plants.
Authentication	This study did not involve plants.
